# The Effect of Dwarfing Interstocks on Vegetative Growth, Fruit Quality and Ionome Nutrition of ‘Fuji’ Apple Cultivar ‘Tianhong 2’—A One-Year Study

**DOI:** 10.3390/plants12112158

**Published:** 2023-05-30

**Authors:** Shuang Li, Yanghong Zhang, Haowei Chen, Boyang Li, Bowen Liang, Jizhong Xu

**Affiliations:** College of Horticulture, Hebei Agricultural University, Baoding 071001, China

**Keywords:** apple, interstock, mineal nutrients, branch, fruit quality

## Abstract

Dwarfing interstocks play an essential role in determining the performance of fruit trees. SH40, Jizhen 1, and Jizhen 2 are widely used dwarfing interstocks in Hebei Province, China. This study examined the influence of these three dwarfing interstocks on vegetative growth, fruit quality and yield, and leaf and fruit macro- (N, P, K, Ca, and Mg) and micro- (Fe, Zn, Cu, Mn, and B) element contents for ‘Tianhong 2’. Five-year-old ‘Fuji’ apple cultivar, ‘Tianhong 2’, on ‘*Malus. Robusta*’ rootstock, was cultivated with SH40, Jizhen 1, or Jizhen2 dwarfing rootstock as an interstock bridge. Jizhen 1 and 2 had more branches and a higher proportion of short branches than SH40. Jizhen 2 had a higher yield, good fruit quality, and higher leaf macro- (N, P, K, and Ca) and micro-element (Fe, Zn, Cu, Mn, and B) contents; Jizhen 1 had the highest leaf Mg content in the growing period. The fruit N, P, K, Fe, Zn, Cu, Mn, and B contents were higher in Jizhen 2. SH40 had the highest fruit Ca content. There were significant correlations in nutrient elements between leaves and fruit in June and July. Comprehensive analysis showed that Tianhong 2 had moderate tree vigor, high yield, good fruit quality, and high mineral element content in leaves and fruits when Jizhen 2 was used as an interstock.

## 1. Introduction

The apple is one of the four most commonly eaten fruits. Dwarfing and “close planting cultivation” are being widely adopted in the apple industry. Dwarfing rootstock is the main route to dwarfing apple. The rootstock can affect the growth, development [1], and vigor of apple trees [2], as well as fruit yield and quality [3,4]. The rootstock also affects apple leaf and fruit mineral content [5,6], which in turn affects the sugar and acid content of the fruit [7]. Although some rootstocks show cold resistance in their place of origin, they often fail to overwinter safely in the main apple-producing areas of northern China, because of the local climatic and ecological conditions. Dwarf interstocks are relatively unsuited to the environmental conditions, but have nevertheless become the main method of dwarfing for close-planting cultivation.

Interstocks can improve yield efficiency, promote early fruit maturation, increase fruit weight [8], induce branching [9], influence fruit quality [10], and affect the synthesis and metabolism of apple trees [11]. Differences in mineral metabolism among interstocks result in differences in tree mineral nutrient contents. Milosevic and Milosevic [12] showed that the interstock modified the content of nutrient minerals in apricot trees. The interstock of apple trees can also block macromolecular nutrient transport, which affects the absorption and utilization of mineral elements [13]. Thus, it is necessary to study the effects of different interstocks on mineral nutrition.

Hebei is one of the main apple-producing areas in China. At present, SH40 is the main interstock in Hebei Province due to its outstanding cold resistance, high yield, and good fruit quality of grafted varieties. Jizhen 1 and 2 are excellent apple dwarf rootstocks bred by Hebei Agricultural University; both are seeding progeny of SH40. Tianhong 2 has good affinity with these interstocks, and is associated with early fruit yield, good fruit quality, and excellent overall performance in Hebei Province [14,15]. However, few studies have examined Tianhong 2 using SH40, Jizhen 1, and Jizhen 2 as interstocks; the mineral contents in leaves and fruit may differ according to the interstock used. Therefore, this study assessed the tree growth, yield, fruit quality, and leaf and fruit mineral nutrient contents of Tianhong 2 when using SH40 and Jizhen 1 and 2 as interstocks in Hebei Province.

## 2. Results

### 2.1. Growth Parameters

**Figure 1:**
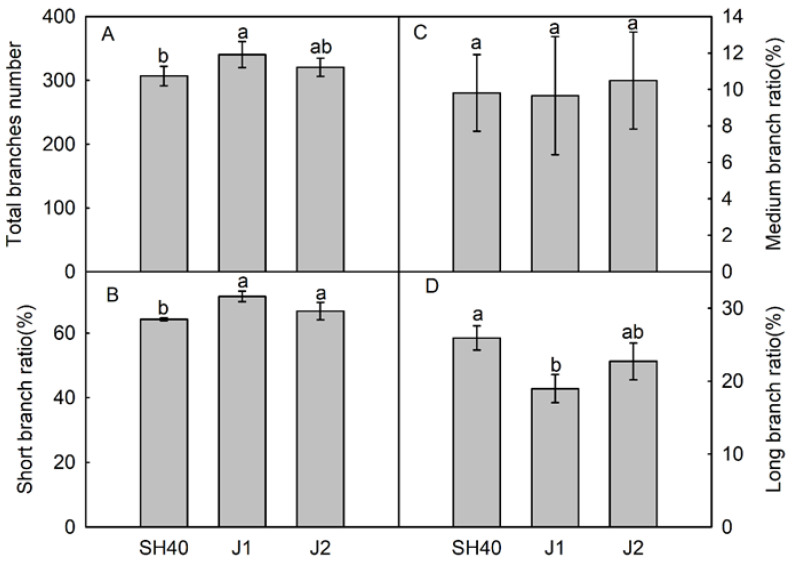
Effects of dwarfing interstocks on the proportion of branch categories. Date are means ± SD (*n* = 4). (**A**) total branches number; (**B**) short branch nratio; (**C**) medium branch raito; (**D**) long branch ratio. Different letters indicate significant differences between treatments, according to one-way ANOVA followed by Tukey’s multiple range test at *P*_0.05_ level.

There were significant differences in branch growth among the interstocks (Figure 1). With Jizhen 1 as the interstock, Tianhong 2 averaged 340.25 branches, of which 71.36% were short; this was significantly higher than the proportion when using SH40 as the in-terstock; Jizhen 2 was second. The proportion of intermediate branches did not differ significantly among the interstocks. The proportion of long branches for Tianhong 2 grown on SH40 was 25.92%, which was significantly higher than that for Jizhen 1 (Figure 1).

### 2.2. Fruit Yield and Quality

**Figure 2:**
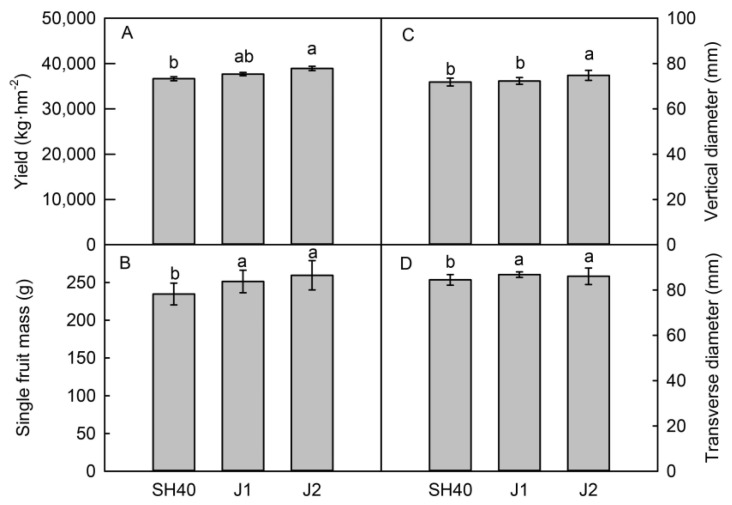
Effects of dwarfing interstocks on fruit size and yield. Date are means ± SD (*n* = 4). (**A**) the yield of fruit; (**B**) the single fruit mass of fruit; (**C**) the vertical diameter of rruit; (**D**) the transverse diameter of fruit. Different letters indicate significant differences between treatments, according to one-way ANOVA followed by Tukey’s multiple range test at *P*_0.05_ level.

**Figure 3:**
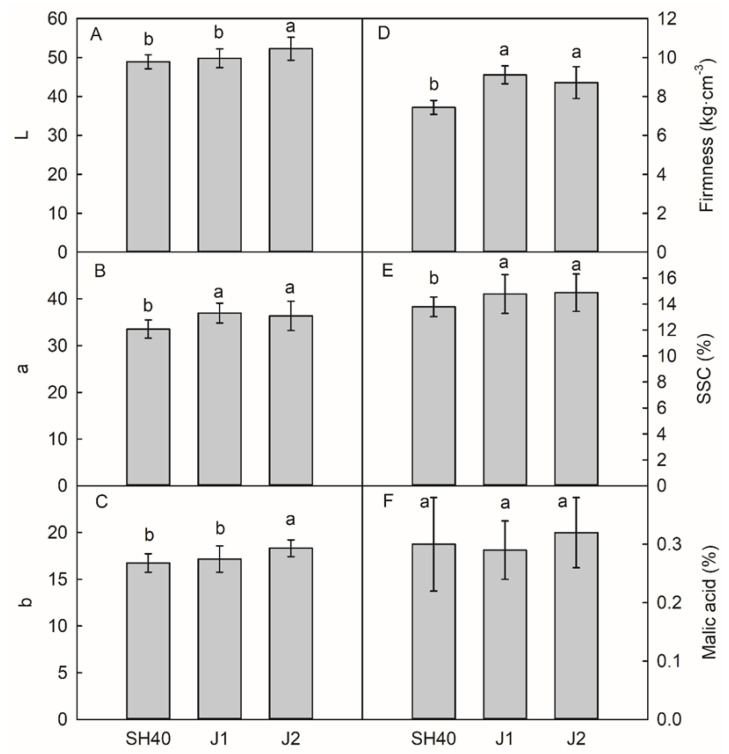
Effects of dwarfing interstocks on fruit quality. Date are means ± SD (*n* = 4). (**A**) L value of apple peel; (**B**) a value of apple peel; (**C**) b value of apple peel; (**D**) fruit firmness; (**E**) fruit soluble solids content; (**F**) fruit malic acid content. Different letters indicate significant differences between treatments, according to one-way ANOVA followed by Tukey’s multiple range test at *P*_0.05_ level.

Different dwarfing interstocks had different effects on the fruit quality of Tianhong 2 (Figure 2 and Figure 3). In 2021, trees on Jizhen 2 had a significant higher yield than trees on SH40 or Jizhen 1, by 6.18% and 3.27%, respectively (Figure 2). Tianhong 2 on Jizhen 1 or 2 had a higher single fruit mass than on SH40. Jizhen 2 had a greater fruit vertical diameter, while Jizhen 1 and 2 had greater transverse diameters, firmness, and SSC contents than those on SH40. There were no significant differences among interstocks in terms of the malic acid content of Tianhong 2. Jizhen 2 had the highest L* and b* values, indicating that the peel was brighter. Jizhen 1 and 2 had higher a* values than SH40; they had good color and a red pericarp (Figure 3).

### 2.3. Fruit Nutrient Concentrations

**Figure 4:**
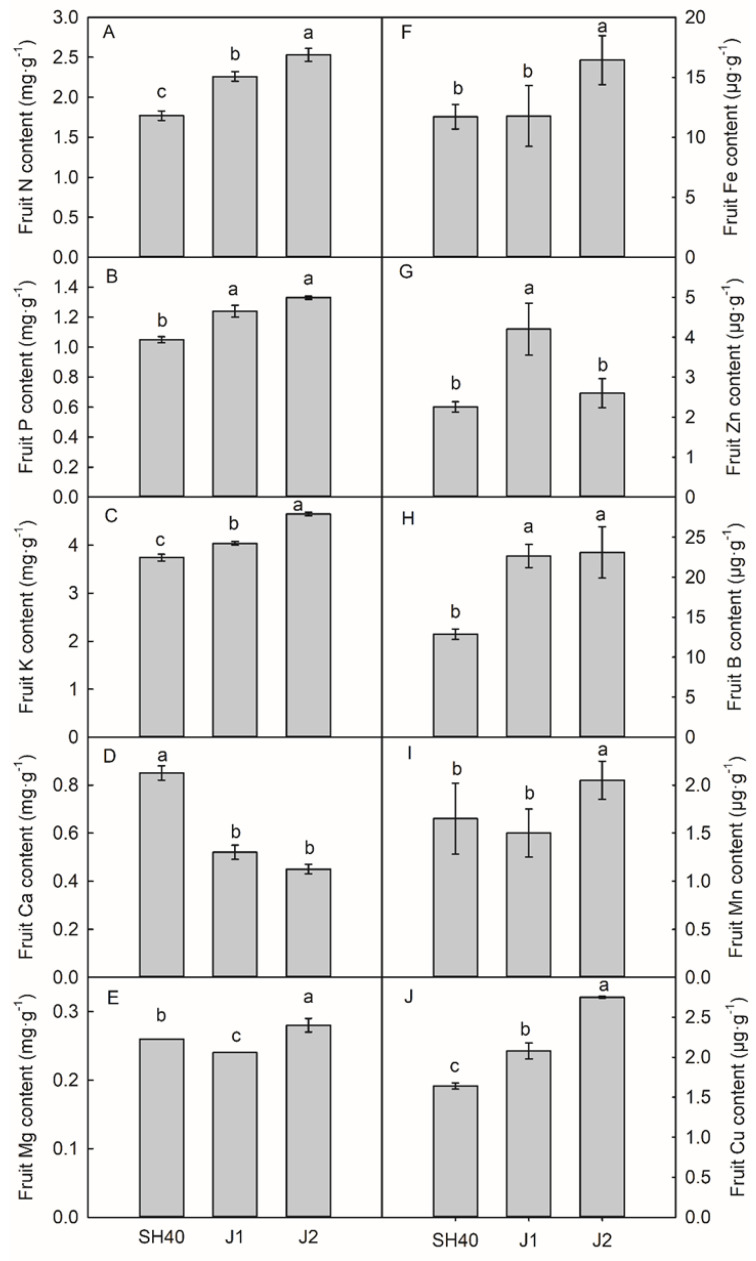
Effects of dwarfing interstocks on concentrations of macroelements and microelements in fruit dry matter. Date are means ± SD (*n* = 3). (**A**) fruit nitrogen content; (**B**) fruit phosphorus content; (**C**) fruit potassium content; (**D**) fruit calcium content; (**E**) fruit magnesium content; (**F**) fruit iron content; (**G**) fruit zinc content; (**H**) fruit boron content; (**I**) fruit manganese content; (**J**) fruit copper content. Different letters indicate significant differences between treatments, according to one-way ANOVA followed by Tukey’s multiple range test at *P*_0.05_ level.

The interstocks influenced the fruit mineral element contents of Tianhong 2. Compared with the other interstocks, Jizhen 2 had the highest fruit N (2.53 g·kg^−1^), P (1.33 g·kg^−1^), K (4.65 g·kg^−1^), Mg (0.28 g·kg^−1^), Fe (16.44 μg·g^−1^), B (23.09 μg·g^−1^), Mn (2.05 μg·g^−1^), and Cu (2.75 μg·g^−1^) contents; SH40 had the highest fruit Ca (0.85 g·kg^−1^) content; and Jizhen 1 had the highest Zn (4.20 μg·g^−1^) content (Figure 4).

### 2.4. Leaf Nutrient Concentrations

**Figure 5:**
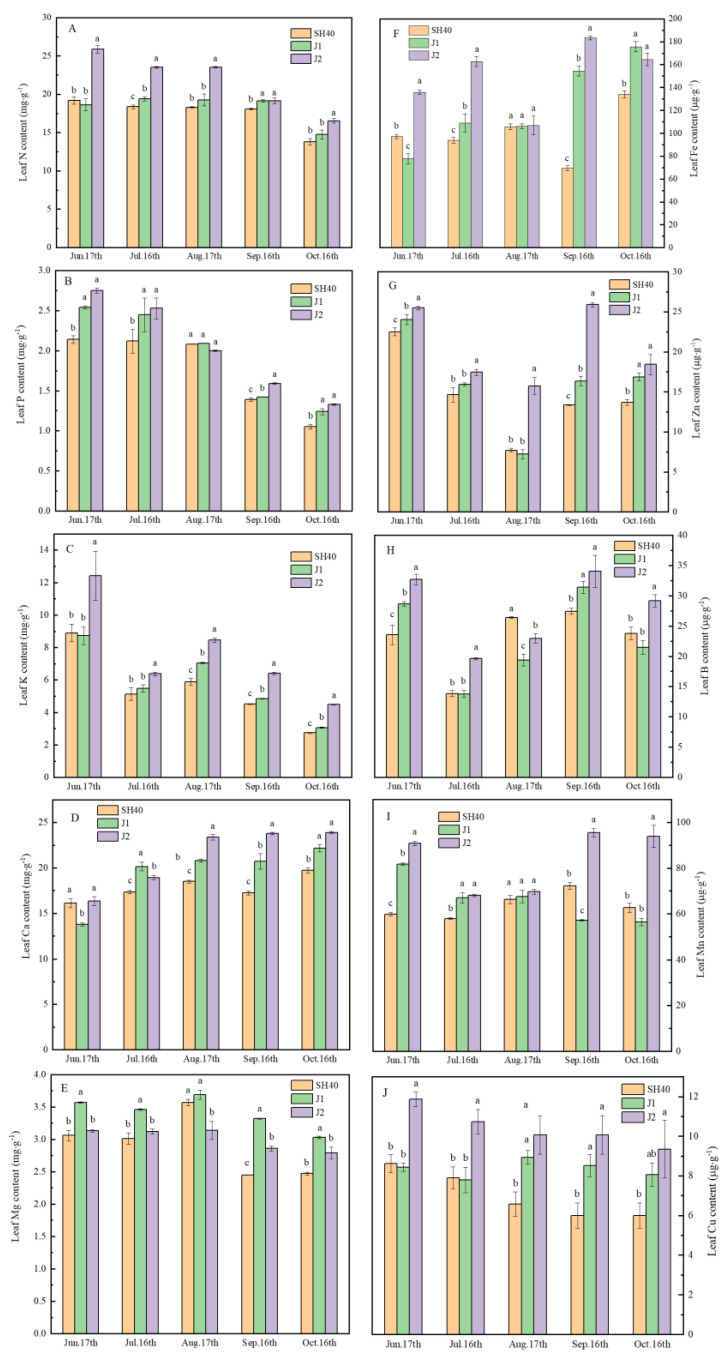
Seasonal variation in concentrations of macro- and microelements in leaf dry matter grown on different dwarfing interstocks. Date are means ± SD (*n* = 3). (**A**) leaf nitrogen content; (**B**) leaf phosphorus content; (**C**) leaf potassium content; (**D**) leaf calcium content; (**E**) leaf magnesium content; (**F**) leaf iron content; (**G**) leaf zinc content; (**H**) leaf boron content; (**I**) leaf manganese content; (**J**) leaf copper content. Unit of measure: mg·g^−1^ DW for N, P, K, Ca, and Mg; μg·g^−1^ DW for Fe, Mn, Cu, Zn, and B. Different letters indicate significant differences between treatments, according to one-way ANOVA followed by Tukey’s multiple range test at *P*_0.05_ level.

The nutrient concentrations of apple leaves on different rootstocks varied significantly depending on the growing period. The Ca levels generally increased from mid-June to mid-October, while the N, P, K, and Mg contents generally decreased (Figure 5). There were significant differences in macronutrient contents among the interstocks (*p* < 0.05). In the five growth periods, the leaf N content of the three interstocks differed significantly. The leaf N content was highest on Jizhen 2, followed by Jizhen 1, and then SH40. During the growth period, the leaf P content remained high on Jizhen 2, while it was lower in leaves on SH40. Similarly, the K content was significantly higher in leaves on Jizhen 2 than SH40. On 17 June, SH40 and Jizhen 2 had higher leaf Ca contents than Jizhen 1, while on 16 July the leaf Ca content was significantly higher on Jizhen 1 than on the other two interstocks. The leaf Mg content was higher on Jizhen 1 than on the other interstocks in each month. Trees grafted on Jizhen 1 appear to have higher leaf Mg contents. The leaf N, P, K, and Ca contents were higher on Jizhen 2 (Figure 5).

The leaf Fe content varied markedly over the study period; the leaf Fe content differed significantly on the three interstocks, but was highest on Jizhen 2 and lowest on SH40 (Figure 5). The leaf Zn and B contents decreased between mid-June and mid-July, and then increased between mid-August and mid-October. Little variation was observed in the Mn and Cu contents. The leaf Fe, Zn, B, Mn, and Cu contents were higher on Jizhen 2. In the five periods, Tianhong 2 on Jizhen 2 had the highest leaf Zn, B, Mn, and Cu contents, while they were lowest on SH40 (Figure 5).

### 2.5. Correlation between the Mineral Element Contents of Leaves and Fruit of Apple Grafted on Different Interstocks

**Figure 6:**
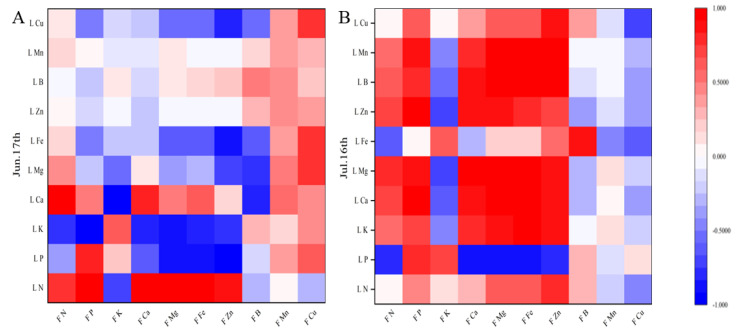
Correlation between the mineral element contents of leaves and fruit of apple grafted on SH40 interstock. L N = Leaf N, L P = Leaf P, L K = Leaf K, L Ca = Leaf Ca, L Mg = Leaf Mg, L Fe = Leaf Fe, L Zn = Leaf Zn, L B = Leaf B, L Mn = Leaf Mn, L Cu = Leaf Cu, F N = Fruit N, F P = Fruit P, F K = Fruit K, F Ca = Fruit Ca, F Mg = Fruit Mg, F Fe = Fruit Fe, F Zn = Fruit Zn, F B = Fruit B, F Mn = Fruit Mn, F Cu = Fruit Cu. (**A**) on 17 June; (**B**) on 16 July.

**Figure 7:**
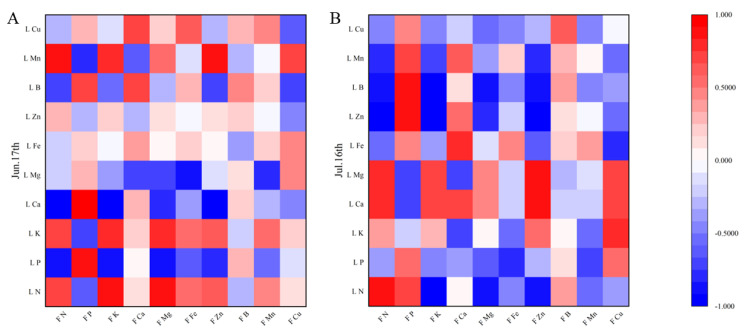
Correlation between the mineral element contents of leaves and fruit of apple grafted on Jizhen 1 interstock. L N = Leaf N, L P = Leaf P, L K = Leaf K, L Ca = Leaf Ca, L Mg = Leaf Mg, L Fe = Leaf Fe, L Zn = Leaf Zn, L B = Leaf B, L Mn = Leaf Mn, L Cu = Leaf Cu, F N = Fruit N, F P = Fruit P, F K = Fruit K, F Ca = Fruit Ca, F Mg = Fruit Mg, F Fe = Fruit Fe, F Zn = Fruit Zn, F B = Fruit B, F Mn = Fruit Mn, F Cu = Fruit Cu. (**A**) on 17 June; (**B**) on 16 July.

**Figure 8:**
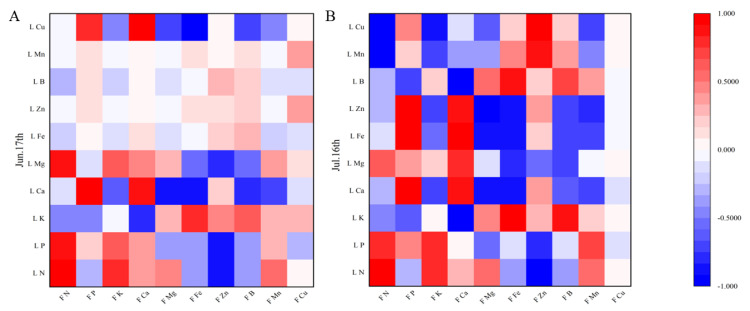
Correlation between the mineral element contents of leaves and fruit of apple grafted on Jizhen 2 interstock. L N = Leaf N, L P = Leaf P, L K = Leaf K, L Ca = Leaf Ca, L Mg = Leaf Mg, L Fe = Leaf Fe, L Zn = Leaf Zn, L B = Leaf B, L Mn = Leaf Mn, L Cu = Leaf Cu, F N = Fruit N, F P = Fruit P, F K = Fruit K, F Ca = Fruit Ca, F Mg = Fruit Mg, F Fe = Fruit Fe, F Zn = Fruit Zn, F B = Fruit B, F Mn = Fruit Mn, F Cu = Fruit Cu. (**A**) on 17 June; (**B**) on 16 July.

Pearson correlation coefficients were calculated between apple leaf and fruit mineral contents in June and July on different interstocks, as a basis for reasonable fertilizer application. There were significant correlations among the nutrient elements (Figure 6, Figure 7 and Figure 8). Fruit N, P, and Ca had highly positive correlations with leaf N, P, and Ca, respectively, while leaf P had negative correlations with fruit Mg, Fe, and Zn.

## 3. Materials and Methods

### 3.1. Plant Material and Growing Conditions

The experiment was conducted at Hebei Agricultural University, Baoding (38°23′ N, 115°28′ E), Hebei, China, which has an elevation of 291 m, 2500 h of annual sunshine, and a mean annual rainfall of 580 mm. ‘Fuji’ apple cultivar ‘Tianhong 2’ apple trees were grafted on three different interstock/rootstock combinations: SH40/*Malus. robusta*, Jizhen 1/*Malus. robusta*, Jizhen 2/*Malus. robusta*, and these were planted in 2016 in a shallow sandy-loam soil. The trees were trained to a free spindle training system at a spacing of 4 m between rows and 1.5 m between trees. The experiment used a completely randomized design, each combination included ten replicates with ten trees (each tree was considered one replication). Consistent cultivation and management measures were adopted.

### 3.2. Growth Parameters

At the end of the growing season (November 2021), the total number of branches and numbers of long (>15.0 cm), intermediate (5.0–15.0 cm), and short (<5.0 cm) branches on each tree were counted, and the proportion of each branch type was calculated.

### 3.3. Fruit Yield and Quality

The weight and quality of apple fruits were assessed in mid-October 2021. Fruits from each rootstock–spike combination were randomly picked 1.5 m above the ground at the outer edge of the tree crown in four directions (east, west, south, and north). A total of 20 fruits per tree were harvested as one biological replicate; there were four biological replicates and 80 fruits were obtained for analysis of fruit quality, and the yield per tree was measured during harvest. The single fruit weight was measured by a balance with a precision of 0.1 g, and the yield in kg·hm^−2^ was calculated. The vertical and transverse diameters of fruits were measured by a digital vernier caliper. A colorimeter (CR-400) was used to determine the L*, a*, and b* values of the fruit at four randomly selected points in the equatorial region, and the average value was calculated. The peel was removed from all four sides of each fruit, and the probe of a GY-1 hardness tester was inserted into the flesh vertically and uniformly to measure firmness. After peeling, two symmetrical pieces of fruit were obtained, and the juice in the flesh of these two pieces was extruded using a simple juice extraction device for measurement of the soluble solids content (SSC; measured using a PAL-1 digital handheld glucose meter). Then, 0.3 mL of juice was added to 50 mL distilled water and the malic acid content was measured.

### 3.4. Nutrient Analysis

Leaf mineral content was measured monthly from June to October, 20 leaves from the middle parts of shoots were selected at random from each tree as one biological replicate, and there were six biological replicates. To measure the fruit mineral content, 20 fruits per tree were selected in mid-October 2021. The collected leaves and fruit samples for each treatment were washed in 0.1 mol·L^−1^ of HCL and rinsed with distilled water, then fixed at 105 °C for 15 min and dried in an oven at 55 °C to a constant weight. After oven drying, the leaf and fruit samples were ground, sieved and mixed. Then, 0.2 g samples were digested with concentrated sulfuric acid and H_2_O_2_, and the N and P concentrations were analyzed in a continuous flow analytical system (AA3; SEAL Analytical, Norderstedt, Germany). Other 0.1 g samples were digested with nitric acid, and other elements were measured by inductively coupled plasma mass spectrometry (ICP-MS, Thermo Fisher Scientific, Waltham, MA, USA). The N, P, K, Ca, and Mg contents are expressed in g·kg^−1^, and the B, Fe, Zn, Cu, and Mn contents in μg·g^−1^, all on a dry weight basis.

### 3.5. Statistical Analysis

The data are presented as the mean ± standard deviation (SD). To detect differences among the groups, the data were subjected to one-way analysis of variance, followed by Tukey’s multiple range test. All statistical analyses were performed using SPSS 20.0 software (IBM Corp., Armonk, NY, USA). A *p*-value < 0.05 was considered significant.

## 4. Discussion

### 4.1. Effects of the Dwarfing Interstocks on Branch Composition and Fruit Yield

As a perennial crop, apple has many types of branches, and the photosynthetic and nutrient transport capacities of the leaves differ among branches, as does their contribution to fruit growth and development [16,17]. The less vigorous the tree, the higher the proportion of short branches, and the greater the nutrient accumulation; more nutrients promote flower bud differentiation and improve yield [18,19,20]. In this study, compared with SH40 and Jizhen 2, short and intermediate branches accounted for 81.02% of the branches on Jizhen 1, and the yield was 33,505.33 kg·hm^−2^. These results concur with Zhang et al. [14,15]. SH40 had fewer short branches and a higher proportion of long branches, which may be due to the excessive growth of apple trees and inhibition of flower bud differentiation due to a lack of nutrients, resulting in lower early yield.

### 4.2. Effects of Dwarfing Interstocks on Apple Quality

With the popularization of apple dwarf close planting, much research has examined the effect of interstocks on apple fruit quality [9]. Fruit size is an important index of fruit quality and is closely related to cell number, cell gap, and cell volume [21]. In our study, Jizhen 1 and 2 had larger fruit. Peel color is also an important index of apple quality, and is affected by genetic factors, the environment, cultivation, and management methods, as well as the nutritional status of the tree itself. Tianhong 2 grafted on Jizhen 1 interstock had a smooth fruit surface and bright skin color. The intrinsic quality difference of apple fruit grafted on different interstocks is mainly reflected in the flavor and texture of the fruit. In our study, different interstocks had different effects on fruit firmness. Jizhen 1 and 2 had greater firmness than SH40. The SSC and malic acid contents are important indicators of the flavor of apple fruit, and different interstocks have different effects on their contents [22]. Jizhen 1 and 2 had higher SSCs than SH40, and Jizhen 2 had the highest malic acid content (0.32%).

### 4.3. Effects of the Dwarfing Interstocks on Ionome Nutrient Concentrations in Fruit Dry Matter

The rootstock not only has significant effects on apple fruit growth and development, but also affects the absorption and transport of mineral elements in apple [4,23]. Different rootstock types have different effects on the absorption and transport capacity of mineral elements in trees [24]. Interstocks affect the content of mineral elements in apple plants [13]. In the present study, Jizhen 2 interstock had higher fruit N, P, K, Mg, Fe, B, Mn, and Cu contents; SH40 had the highest fruit Ca content; and Jizhen 1 had the highest Zn content.

### 4.4. Effects of Dwarfing Interstocks on Ionome Nutrient Concentrations in Leaf Dry Matter

As an important part of fruit trees, rootstock not only regulates the growth and development of the aboveground part of the tree, but also affects the absorption and utilization of its mineral elements. The leaves show the response of the tree to mineral elements, and their mineral element content is often used to determine tree nutrient levels [25]. The N, P, K, Ca, Mg, Fe, Mn, and Zn contents in leaves affect apple yield [26,27,28]. We found that Jizhen 2 resulted in the highest leaf N, P, K, Ca, Fe, Zn, Mn, and Cu contents, while Jizhen 1 had the highest leaf Mg content in the five periods. Their yields were also relatively high. The mineral element contents in SH40 leaves were lower than the other interstocks. The leaf N and Ca contents are related to fruit size [29] and firmness [30]. The SSC is related to the leaf K, Ca, Fe, and Zn contents. Fruit acidity is positively influenced by K and P levels in the leaf [31]. Our results showed that Jizhen 2 had high mineral–element contents, which might explain its better fruit quality.

The absorption of different minerals by leaves varies throughout the growing season. Zarcinas et al. [32] and Nachtigall and Dechen [33] reported that leaf N, P, and K contents decreased as the apple tree vegetative cycle progressed, while the leaf Ca content increased with leaf age. Cruz et al. [34] observed little variation in leaf Mn and Cu contents during the growing season. Our results are largely consistent with these reports. Apple fruits enlarge from June to August; nutrient elements in leaves are transferred to the fruit, resulting in a decrease in these minerals in leaves. At this time, fertilization can improve the photosynthetic efficiency of leaves, promote nutrient accumulation, and meet the nutritional requirements for fruit enlargement. Trace elements are difficult to transfer out of leaves, even if large amounts are absorbed during the fruit expansion period; therefore, supplemental trace element spraying is required. Thus, nutrient accumulation during tree storage should be examined to ensure that the nutritional needs of growing fruit are met.

### 4.5. Correlations between Leaf and Fruit Mineral Elements

Leaves are important for plant nutrient manufacture, flower bud differentiation, and the storage of the nutrients required for fruit growth and development. Leaf and fruit mineral element analysis can provide a comprehensive picture of the nutritional status of trees. In this study, fruit N, P, and Ca had highly positive correlations with leaf N, P, and Ca, respectively, in agreement with Fallahi et al. [35]. Leaf P was negatively correlated with fruit Mg, Fe, and Zn. The changes in mineral elements in leaves and fruits differed among interstocks, possibly due to differences in the degree of synergy or antagonism between mineral elements. Certain elements with functions in plant physiological and biochemical processes showed relatively large correlations between apple leaves and fruit. Therefore, during the cultivation process, plant–mineral contents can be precisely regulated through correlation analysis of mineral elements in leaves and fruit.

## 5. Conclusions

When using Jizhen 2 interstock, Tianhong 2 had moderate tree vigor, high yield, good fruit quality, and high mineral–element contents in leaves and fruit. Jizhen 2 is a suitable dwarfing interstock for Tianhong 2 and should be subject to further tests and evaluations.

## Data Availability

The data can be obtained from the authors upon request.
